# Evaluation of organochlorine pesticide residues in *Beta* *vulgaris*, *Brassica* *oleracea*, and *Solanum* *tuberosum* in Bloemfontein markets, South Africa

**DOI:** 10.1002/fsn3.2375

**Published:** 2021-07-29

**Authors:** Nthabiseng Motshabi, Somandla Ncube, Mathew Muzi Nindi, Zenzile Peter Khetsha, Ntsoaki Joyce Malebo

**Affiliations:** ^1^ Department of Life Sciences Central University of Technology Bloemfontein South Africa; ^2^ Department of Chemistry Sefako Makgatho Health Sciences University Pretoria South Africa; ^3^ Department of Chemistry The Science Campus University of South Africa Florida Park South Africa; ^4^ Department of Agriculture Central University of Technology Bloemfontein South Africa

**Keywords:** GC‐HRT/MS, health risk assessment, pesticides, QuEChERS, vegetables

## Abstract

This study evaluated the level of selected pesticide residues in the staple vegetables; *Brassica* *oleracea* *var*. *capitata* (cabbage), *Beta* *vulgaris* *var*. *cicla* (Swiss chard), and *Solanum* *tuberosum* (potato) from fresh produce markets in the city of Bloemfontein, South Africa. A QuEChERS extraction method was used followed by quantitation using GC‐HRT/MS. The pesticide residues were detected in levels lower than the recommended Maximum Residue Levels ranging from not detected to 121.6 ng/kg recorded for heptachlor in cabbage samples. Cabbage was generally susceptible to pesticide residue accumulation with the average total concentration for different markets at 222 mg/kg. The pesticide residues were predicted to be from recent applications but their existence within guideline limits indicated that their use in vegetable farming was within the FAO/WHO recommended good agricultural practices. While the current situation points that consumption of the vegetables in the province poses limited health concerns due to organochlorine pesticides, the unmonitored use of products containing these compounds may result in elevated levels. Continued monitoring and a call for the South African legislature to revise its regulations of the Fertilizers Act to reflect the current international laws on pesticides management is recommended.

## Graphical abstract

The study presents a health risk assessment on consumption of three common vegetables: *Brassica* *oleracea* *var*. *capitata* (cabbage), *Beta* *vulgaris* *var*. *cicla* (Swiss chard), and *Solanum* *tuberosum* (potato) from fresh produce markets in the city of Bloemfontein, South Africa. A QuEChERS extraction method was used followed by quantitation using GC‐HRT/MS. The pesticide residues were detected in levels lower than the recommended maximum residue levels ranging from not detected to 121.6 ng/kg recorded for heptachlor in cabbage samples.

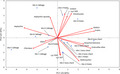

## 1 INTRODUCTION

Food safety has become a primary public concern worldwide, due to pesticide residues in food resulting from direct application of pesticides to crops. Pesticides are mainly applied in the farming industry to control vectors that reduce the quantity and quality of farm produce. The pesticides are sprayed directly on the crops/vegetables and the soil surface. Some pesticide residues are persistent and can remain on the surface of the crops and/or absorbed from soil into the plant system. Consumption of farm produce, therefore, becomes the primary source of exposure of the general population to pesticides. The pesticide metabolites in the human body have been linked to a wide variety of health effects, ranging from acute to chronic toxicity; such as cancer, endocrine disruption, and neurological effects (Taiwo, 2019).

Due to the side effects linked to pesticides, it has become crucial to control the use of pesticides within regulatory limits. However, it is noted in literature that while every country has its own regulations and laws regarding pesticides, governments in developing countries do not adequately address the unsafe use of pesticides (Abubakar et al., 2015). In this regard, elevated levels of pesticide residues may occur in crops due to various factors such as irresponsible handling, storage, and transport of pesticides as well as incorrect application techniques and poorly maintained or inappropriate spraying equipment. Due to such inappropriate practices, farmers increase the health risk of pesticide use.

Vegetables are a major source of vitamins, minerals, antioxidants, and other essential nutrients. A high intake of vegetables is encouraged to prevent negative consequences due to vitamin deficiency and to reduce the incidence of major diseases such as cancer, cardiovascular diseases, and obesity (Hounsome et al., 2008). However, the physiology of most vegetables makes them susceptible to pesticide contamination compared with grain crops. Various studies on the presence of organochlorine pesticide residues in vegetables have been done across the world (Olisah et al., 2019) but recently, studies are mainly in developing countries (Ali et al., 2014; Elibariki & Maguta, 2017; Taiwo, 2019). In most developed countries, the use of organochlorine pesticides is banned in favor of alternative safer compounds. In this regard, health risk assessment studies have focused on the permitted less‐persistent pesticide products especially organophosphorus, carbamates, nicotinoid, and pyrethrins (Chau et al., 2020; Fonseca et al., 2019; Lin et al., 2018; Montiel‐León et al., 2019; Narenderan et al., 2020). However, for developing countries especially Africa, there are no measures to ensure farmers stick to prescribed pesticide products and application of banned products continues unabated (Adewunmi & Fapohunda, 2018). In this regard, monitoring studies on farmed food products in these areas is essential. Notably, most of these organochlorine pesticide residues in developing countries have been found in elevated concentrations above international guidelines (Adewunmi & Fapohunda, 2018; Elibariki & Maguta, 2017; Lehmann et al., 2017; Nuapia et al., 2016; Taiwo, 2019). These findings highlight the need for continuous monitoring and health risk assessment to protect the human population from pesticide poisoning from food sources.

This study, therefore, seeks to evaluate the presence of selected pesticide residues in vegetables from fresh produce markets in Bloemfontein, South Africa. Three staple vegetables common within the South African markets (Shackleton et al., 2010); *Brassica* *oleracea* *var*. *capitata* (cabbage), *Beta* *vulgaris* *var*. *cicla* (Swiss chard), and *Solanum* *tuberosum* (potato) were analyzed for the presence of 16 selected organochlorine pesticide residues using the Quick Easy Cheap Effective Rugged and Safe (QuEChERS) extraction technique followed by determination/quantitation using gas chromatography coupled to high‐resolution time‐of‐flight mass spectrometry (GC‐HRT/MS). The QuEChERS method is the most effective method in analysis of pesticides in food sources (Alcântara et al., 2019; Lehotay et al., 2010; Musarurwa et al., 2019; Santana‐Mayor et al., 2019). Human risk assessment studies were also done to determine any potential risks to an average person consuming the vegetables. Consequently, the results of this nature might be used in future when done on a larger scale to design future control programs for this area and in taking preventive actions to minimize human health risk if necessary.

## 2 MATERIALS AND METHODS

### 2.1 Study area and sampling technique

Sampling was done in April 2019 from four major vegetable markets in Bloemfontein, Free State Province, South Africa. The sampling sites were labelled as market A, market B, market C, market D, and market E. Markets B and D were the same brands but in different locations within the city. The selected vegetables purchased from fresh produce markets were cabbage, Swiss chard, and potato. A total of 15 samples (five heads of cabbage, five bunches of Swiss chard and five potatoes) were purchased randomly from the fresh produce supermarkets. Cabbage, Swiss chard, and potato samples were placed in sterile polythene bags and put in an ice chest box packed with ice and transported to the laboratory. In the laboratory, the samples were washed with deionized water and then stored in a fridge at –4˚C until further processing.

### 2.2 Chemicals and standards

HPLC‐grade ethyl acetate, acetonitrile, formic acid, and acetone were purchased from Sigma‐Aldrich. All the pesticide residues were of analytical grade and purchased in powder‐form separately from Supelco. A QC Solution, AOAC Method 2007.01 containing triphenyl phosphate (500.4 ± 2.5 μg ml^‐1^) and an HPLC and GC Internal Standard, AOAC Method 2007.01 containing parathion‐d10 (diethyl‐d10) and alpha‐BHC‐d6 (alpha‐HCH‐d6) with certified concentrations of 1,002 ± 5 μg/ml and 1,001 ± 5 μg/ml were purchased from Agilent Technologies. The QuEChERS kits for the AOAC Method 2007.01 were from Restek Corporation. The kits consisted of packets of pre‐weighed extraction salts, each containing 6 g anhydrous magnesium sulfate (MgSO_4_) and 1.50 g anhydrous sodium acetate. The prepacked centrifuge tubes (15 ml) each containing 150 mg of magnesium sulfate, 50 mg primary secondary amine (PSA), and 50 mg graphitized carbon black were also purchased from Restek Corporation.

### 2.3 Sample preparation and extraction

The 15 samples from each market were prepared by using a knife to cut off all unwanted plant parts, including stalks. This was followed by washing off soil and dirt with running water from the tap. A potato peeler was used to peel potatoes. Then, followed the cutting of samples into small portions (1–3 cm diameter). Each set of vegetable samples was ground and homogenized into a soupy mixture using a kitchen blender. The homogenized samples were portioned into triplicates of ±200 g and placed in polyethene plastic bags. Each sample was immediately subjected to QuEChERS extraction for isolation of pesticide residues. The extraction and clean‐up method used were conducted according to the manufacturer's instructions for Q‐sep QuEChERS Extraction kits for the AOAC 2007.01 method, with acetate buffering. The general procedure involved placing 15 g of the homogenized sample (cabbage, Swiss chard or potato) in a 50 ml centrifuge tube. Then, 15 ml of 1% (v/v) acetic acid in acetonitrile was added as an extraction solvent. This was followed by adding 6 g of magnesium sulfate and 1.5 g of sodium acetate to enhance the extraction process by separating the organic phase from the water component of the sample. The sample was mixed using a vortex mixer followed by centrifuging at 1,500 *g* for 1 min. Then, the supernatant was removed for clean‐up, and the dispersive solid‐phase extraction clean‐up was performed to remove organic acids, excess water, and other components with a combination of PSA sorbent and magnesium sulfate.

The 8 ml supernatant was transferred into a 15 ml centrifugal tube. Then, 400 mg PSA and 1,200 mg magnesium sulfate were added to all vegetable samples, and 400 mg graphite carbon black for Swiss chard for removing co‐extractants such as sugars and fatty acids. The extract was shaken for 30 s and then centrifuged for 1 min at 1,500 rpm to separate the solid material. Approximately 4 ml of the supernatant was then filtered through a 0.45 mm PTFE filter. The supernatant was evaporated using a slow stream of nitrogen gas and reconstituted in 1 ml hexane for the analysis.

### 2.4 Pesticide residue analysis

The analysis was performed using a Pegasus GCxGC‐HRT/MS 4D from LECO Ultra, equipped with an autosampler (Agilent Technologies, Palo Alto), and interfaced to a LECO Pegasus^®^ HRT high resolution time‐of‐flight mass spectrometer with electron ionization ion source (LECO).

The separation was achieved in one‐dimensional mode on a Rxi‐5Sil MS column with dimensions 30 m × 0.25 mm i.d. × 0.25 μm *df* (Restek Corp) using helium carrier gas at a flow rate of 1 ml/min. The GC oven temperature program was initially set at of 50°C for 2 min. The temperature was then increased to 160°C at 10°C/min and held for 1 min, then ramped up to 280°C at 5°C/min and held for 2 min. The secondary column and the modulator were treated as a transfer line and their temperature set at 285°C and 295°C throughout the entire run, respectively. The total GC run time was 42 min. The inlet temperature was 250°C while that of the transfer line was 300°C.

### 2.5 Health risk assessment

Health risk estimations were calculated based on an integration of pesticide analysis data and exposure assumptions. Potential human health risks were predicted through the comparison of estimated daily intake (EDI) to the established acceptable daily intake (ADI). The EDI value of each pesticide residue was calculated based on the arithmetic mean concentration of each pesticide residue, the food consumption rate, and the average body weight (60 kg) using Equation 1. The consumption rate for vegetables in South Africa is estimated at 0.235 kg/day per person (Vorster et al., 2013). The chronic consumer health risk, referred to as the hazard quotient (HQ), was calculated as percentage ratio between EDI and ADI using Equation 2. The HQ indicates an unacceptable risk when it is higher than 100%.

(1) $$EDI=\;C_{A}\;\times \;fc/bw$$

where EDI is the estimated daily intake (mg kg‐bw day^−1^) and $C_{A}$ is the average pesticide residue concentration (mg/kg), *fc* is the food consumption rate (kg/day) and *bw* the average body weight (kg)

(2) HQ=EDI/ADI×100%

where HQ is the hazard quotient and ADI is the acceptable daily intake of a pesticide residue (mg kg‐bw day^‐1^)

### 2.6 Quality control

A 100 mg/L mixed stock solution was prepared by weighing, mixing, and dissolving selected pesticide residues in acetonitrile. The stock solution was stored at –4°C and only removed to prepare a 10 mg/ml working standard. This was then used in preparation of calibration and spiking standards. Calibration of the GC‐HRT/MS was done using seven standard solutions in the 0.01–5 ng/ml concentration range. Fragmentation patterns showing exact masses of the base ion and two confirmation ions accurate to five decimal places (Table 1) were used in relation to retention times for accurate identification of targets compounds from chromatograms. For recoveries, the potato vegetable samples were spiked at three concentration levels: namely 2, 10, and 100 ng/kg. These spiking levels were approximately at 3, 10, and 100 × LOQ for most pesticide residues. In this regard, 2 ng/kg was below all recorded concentrations except for those not detected/quantified, the 10 ng/kg values were within the concentration ranges while 100 ng kg was above the concentration limits excepts for heptachlor and endosulfan ether. In addition, QC Solution, AOAC Method 2007.01 consisting of triphenyl phosphate was added to the samples before QuEChERS extraction at 10 × LOQ. HPLC and GC Internal Standard for AOAC Method 2007.01 consisting of parathion‐d10 (diethyl‐d10) and alpha‐BHC‐d6 (alpha‐HCH‐d6) was used as the internal standard and spiked to extracts at 10 × LOQ.

**TABLE 1:** Method validation parameters

Pesticide residue	Retention time (min:s)	Exact mass	*R* ^2^	MDL (ng/kg)	Accurate base ion	Accurate confirmation ions	Recovery (% ± *SD*)
o.p'‐DDT	29:37	351.914688	.9993	0.84	235.00744	235.00740, 165.06980	99 ± 7.27
o.p'‐DDD	28:15	317.953661	.9854	0.21	235.00776	165.07013, 237.00481	98 ± 9.5
p.p'‐DDE	26:44	315.938011	.9975	0.36	245.99944	317.93412, 247.99672	91 ± 17.0
Aldrin	23:08	361.875717	.9961	0.51	66.04653	262.85674, 264.85378	89 ± 14.0
Endrin	27:38	377.87063	.9938	1.6	81.03362	79.05436, 262.85625	87 ± 5.7
Dieldrin	26:53	377.87063	.9886	0.86	79.05432	81.03365, 82.04144	91 ± 10.4
Endrin aldehyde	28:33	377.87063	.9941	0.86	67.05437	344.89825, 249.84875	101 ± 15.7
Chlordane	30:48	405.797771	.9972	0.63	372.82522	374.82230, 376.81929	105 ± 6.3
Chlorbicyclen	29:25	393.797771	.9857	0.86	271.80966	273.80679, 228.89546	89 ± 8.5
Heptachlor	26:01	369.821095	.9791	0.63	100.00743	271.80963, 273.80678	94 ± 13.9
Heptachlor epoxide	24:33	385.816008	.9919	0.71	81.03359	352.84364, 354.84073	79 ± 5.5
Methoxychlor	31:39	344.013763	.9710	0.38	227.10675	238.09897, 274.07544	96 ± 11.4
Endosulfan ether	28:01	339.85498	.9860	1.6	69.03357	240.89552, 238.89828	101 ± 7.27
Lindane	19:10	287.860065	.9963	0.40	181	180.93728, 218.91094	87 ± 7.01
Hexachlorobenzene	18:08	281.813116	.9906	1.7	283.80959	285.80674, 281.81258	67 ± 10.4
Dicofol	31:45	367.909603	.9768	1.0	138.99453	110.99961, 140.99164	88 ± 14.3

Abbreviation: MDL, method detection limit.

## 3 RESULTS AND DISCUSSION

### 3.1 Concentration levels of pesticide residues in vegetable samples

Out of the 16 selected pesticide residues, only dicofol and endrin aldehyde could not be detected in the vegetables (Table 2). The concentrations of dichlorodiphenyltrichloroethane (DDT) and its derivatives, dichlorodiphenyldichloroethane (DDD) and dichlorodiphenyldichloroethylene (DDE) in all the vegetables, were very low ranging between 6.7 and 14.3 ng/kg. These concentration levels among vegetables and markets were not significantly different at 95% confidence interval. DDT is banned in South Africa (DAFF, 2017), but it is still applied in mosquito‐infested areas in South Africa, especially the Limpopo and KwaZulu Natal provinces where its impact in controlling mosquitoes outweighs its human health impacts (Van et al., 2010). However, its detection in the central province of the Free State Province might be related to both past and present agricultural activities with the DDE/DDT and DDD/DDT ratios ranging between 0.8 and 1.5 implying that the source was inconclusive. While DDT and its derivatives remain persistent in the environment, their low concentration in the vegetables observed in the current study may indicate a decline in their presence in the environment. No previous studies have been done in this study area, and continuous monitoring is recommended. Currently, the population's exposure to DDT and its residues through farmed vegetables is limited. Compared with other studies, the current study has shown that presence DDT and its derivatives in farmed vegetables is relatively low in this province. For example, in the KwaZulu Natal Province within South Africa, Buah‐Kwofie et al. (2019) reported high concentrations of DDT, DDD and DDE in the 5.1–19 ng/g in spinach, peanut, onion, and lettuce samples while Nuapia et al., 2018 recorded concentrations between 61.05 and 125.87 µg/kg in cabbage samples from open markets in Johannesburg, South Africa and Kinshasa, Democratic Republic of Congo (Nuapia et al., 2016). In Togo, the concentrations were up to 643 ng/kg in cabbage and 527 and 681 ng/kg in tomato and lettuce samples, respectively (Kolani et al., 2016). In Tanzania, a review article has noted that DDT and its derivatives also exist in the µg/kg scale in food crops and vegetables (Elibariki & Maguta, 2017). These results were a confirmation that DDT‐containing products are still in use in African countries.

**TABLE 2:** The level of pesticide residues (mg/kg‐w/w) in cabbage, Swiss chard and potato samples; *n* = 3, RSD < 17%

Pesticide residue	MRL (mg/kg)	Mean pesticide residue concentration (ng/kg)														
		Market A			Market B			Market C			Market D			Market E		
		Cabbage	Swiss chard	Potato	Cabbage	Swiss chard	Potato	Cabbage	Swiss chard	Potato	Cabbage	Swiss chard	Potato	Cabbage	Swiss chard	Potato
o.p'‐DDD	0.2	8.4	8.7	9.3	8.2	9.0	9.0	9.1	9.1	7.8	8.8	9.3	6.7	8.6	9.4	9.3
p.p'‐DDE	0.01	9.6	7.5	7.7	7.7	9.1	8.0	8.6	9.0	8.0	8.0	8.2	8.3	7.0	7.8	14.3
o.p'‐DDT	0.2	8.4	8.7	9.3	8.2	9.0	9.0	9.1	9.1	8.8	8.8	9.3	6.7	8.6	9.4	9.3
∑DDTs	–	26.4	24.9	26.3	2.41	27.1	26.0	26.8	27.2	24.6	25.6	26.8	21.7	24.2	26.6	32.9
Aldrin	0.05	11.7	9.3	6.1	10.5	10.1	6.0	10.6	9.5	nd	8.7	9.5	5.7	8.8	8.0	7.1
Endrin	0.05	8.0	nd	7.9	6.9	10.8	7.5	8.2	8.5	nd	nd	9.1	9.9	7.1	7.5	nd
Dieldrin	0.05	nd	nd	7.9	6.9	nd	7.5	8.2	nd	nd	nd	nd	9.9	7.1	nd	nd
Endrin aldehyde		nd	nd	nd	nd	nd	nd	nd	nd	nd	nd	nd	nd	nd	nd	nd
∑drins	–	19.7	9.3	21.9	24.3	20.9	21	27	18	nd	8.7	18.6	25.5	23.0	15.5	7.1
Chlordane	0.02	9.9	nd	7.7	nd	50.7	7.7	8.9	7.2	8.4	nd	nd	8.3	16.2	nd	13.5
Chlorbicyclen	0.01	11.8	nd	10.6	11.0	nd	11.2	10.3	nd	nd	nd	nd	11.3	9.9	nd	nd
Heptachlor	0.05	121.6	28.7	18.5	nd	19.9	20.1	23.2	18.6	18.2	19.5	19.2	18.6	116.4	18.1	22.3
Heptachlor epoxide	0.05	13.1	10.9	11.5	11.3	11.3	12.6	13.0	10.8	11.3	12.7	11.0	11.9	12.2	11.2	17.7
Methoxychlor	0.05	62.1	nd	104.5	nd	89.0	nd	nd	nd	nd	77.0	83.8	nd	75.9	97.4	78.4
∑chlors	–	218.5	39.6	152.8	22.3	170.9	51.6	55.4	36.6	37.9	109.2	114	50.1	376.6	126.7	131.9
Endosulfan ether	0.05	9.9	nd	60.4	8.9	85.4	75.0	17.2	99.2	78.7	8.1	32.7	73.3	17.3	100.9	48.4
α‐Lindane	0.01	10.4	nd	nd	nd	nd	nd	2.1	nd	nd	5.4	nd	5.4	3.5	nd	10.4
Hexachlorobenzene	0.01	4.0	2.3	nd	1.6	2.9	19.0	4.2	nd	2.90	2.1	nd	2.7	3.5	nd	6.8
Dicofol	–	nd	nd	nd	nd	nd	nd	nd	nd	nd	nd	nd	nd	nd	nd	nd
∑other	–	24.3	2.3	60.4	10.5	88.3	94.0	23.5	99.2	81.6	15.6	32.7	81.4	24.3	100.9	65.6
∑Total	–	288.9	76.1	261.4	81.2	307.2	192.6	132.7	181.0	144.1	159.1	192.1	178.7	448.1	269.7	237.5

Abbreviations: MRLs, Maximum Residue Limit; nd, not detected.

Generally, the highest concentrations for pesticide residues were recorded for chlors (Table 2). Heptachlor was the highest reaching 121 ng/kg in cabbage samples from Market A. Chlordane was also relatively high reaching 50.7 ng/kg in Swiss card. Chlordane was banned in 2005 in South Africa (DAFF, 2017). It is a highly persistent organic pollutant and remains readily detectable in the environment, which may be the reason why it is still found in crops and vegetables. Additionally, chlordane also exists as a degradation product of heptachlor (ATSDR, 2007). Notably, cabbage was generally susceptible to pesticide residue accumulation with the total targeted chlors reaching 376.6 ng/kg in Market E. The concentration of each of the chlors in the vegetables and the markets was statistically different except for heptachlor epoxide which ranged between 10.8 and 17.7 ng/kg. A comparison of the heptachlor and heptachlor epoxide concentrations for vegetable samples gave ratios between 1.5 and 9 indicating that the presence of chlors is from recent inputs. In South Africa, the use of heptachlor was only banned in 2016 (DAFF, 2017).

The targeted drins were detected in very low concentrations (≤11.7 ng/kg) with endrin aldehyde below detection limits in all samples while dieldrin was detected in only six of the 15 samples from different markets. Diendrin was also not detected in most samples and where detected, it was still lower than endrin. The aldrin/diendrin ratio was therefore always <1 implying recent inputs into the environment. This implied that pest control products containing the drins are still used in farmlands from which the vegetables are sourced. In South Africa, aldrin was only banned in 2016, which would explain this prediction (DAFF, 2017). Elsewhere, adrins have been detected in very high concentrations in cabbage samples in Johannesburg while not detected in those from Kinshasa (Nuapia et al., 2016). Among other pesticide residues included in the study, endosulfan ether was also high especially in Swiss chard reaching 99.2 and 100.9 ng/kg in markets C and D, respectively, while α‐lindane and hexachlorobenzene were not detected in some samples.

It should be emphasized that detection of a pesticide residue does not mean application of pesticide products in which that residue is labelled. A challenge with pesticide residues is related to the purity of their technical grade standards. For example, technical‐grade heptachlor and that of its metabolite, chlordane contain a proportion (~10%) of each other (ATSDR, 2007). Furthermore, heptachlor epoxide is not commercially available but exists in the environment and in organisms as an oxidation by‐product of heptachlor (ATSDR, 2007; Bandala et al., 2006; Purnomo et al., 2013). Therefore, a pesticide product indicating presence of one residue is likely to contain other residues.

Importantly, the concentration of all targeted pesticide residues reported in the current study (Table 2) was below the permissible maximum residue limits (MRLs) in vegetables set by FAO/WHO (FAO, 2016). The MRL is the maximum concentration of a pesticide residue recommended by the Codex Alimentarius Commission to be legally permitted in or on food commodities and animal feed. Exposure to a particular pesticide below the health safety limit is considered safe. The implication of our results is that the use of pesticides in vegetable farming in the Free State Province is within the FAO recommended good agricultural practices (FAO, 2012). However, the detection of some banned pesticide residues may be related to both past and present usage. It is therefore difficult to conclude whether the pesticide residues are from the past and exist due to their persistent nature or farmers still using products containing these residues. However, we have used the residue/by‐product ratios to predict that the pesticide residues are from recent sources, probably application of products containing these residues. Evidence of farmers' incompliance in developing countries has been raised elsewhere (Elibariki & Maguta, 2017; Ozcan & Balkan, 2017). More studies are therefore required to trace the true sources of organochlorine pesticide residues, whether they are from the past or are still in use in farmlands.

### 3.2 Quality control

Quality control parameters are given in Table 1. The calibration curves showed good linearity with coefficient of determination (*R*²) values ranging between .9710 and .9993. Recoveries of the pesticide residues were within the range of the typical acceptance criteria (70%–120%) for quantitative regulatory except for hexachlorobenzene whose average was 67%. The current recoveries are similar to those reported for QuEChERS by other studies including 73%–118% (7), 75.9%–108.2% (10), 80.6%–118.3% (31), and 73%–106% (23). The RSD values for recoveries were in the acceptable range of 6%–18.7%. However, no correction was done in the quantitation of hexachlorobenzene. The precision of the reported concentrations for each vegetable sample was consistently <17% RSD. The QuEChERS‐GC‐HRT/MS method gave pesticide residue detection limits in vegetables in the part per trillion scale ranging from 0.21 to 1.7 ng/kg.

### 3.3 Principal component analyses on vegetables and sites

**FIGURE 1:**
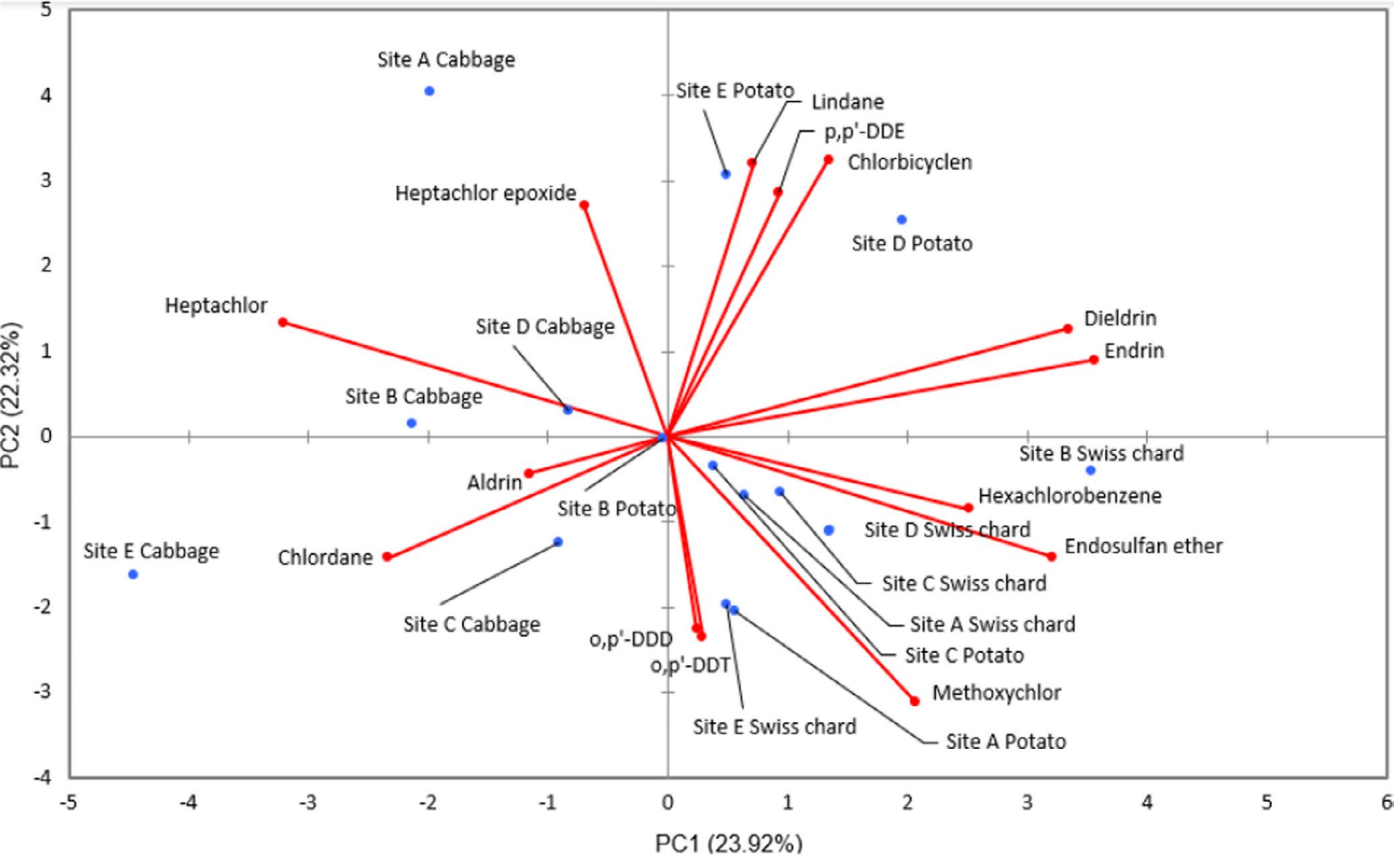
Principal component biplot showing variation between pesticide residues accessions by vegetable and site traits

Principal component analysis (PCA) was performed on R‐Studio version 3.6.1 to find patterns in the distributions of the 16 pesticide residues among 15 vegetable samples obtained from different sampling sites (Figure 1). The input data were partitioned such that 20% as testing data, and 80% was used as training data. The advantage of PCA over scatter plots and cluster diagrams in analysis of large amounts of data is its ability to counteract multicollinearity problems associated with high correlations among independent variables (Naangmenyele et al., 2020). The PCA results show that cabbage was generally susceptible to pesticide residue accumulation which correlated with the average total concentration for different markets of 222 mg/kg compared with 205 and 200 mg/kg for Swiss chard and potato samples, respectively. In addition, the three most common chlor residues, which also exist as degradation products of each other (chlordane, heptachlor, and heptachlor epoxide) (ATSDR, 2007; Purnomo et al., 2013) were highest in cabbage samples. Swiss chard also forms a unique group (positive for PC1 and negative for PC2) characterized by absence of dieldrin, chlorbicyclen and α‐lindane in all markets. These results might indicate that most vegetables from all the fresh produce markets have similar sources or sources with similarities in the farming practices in terms of pest control. Generally, potato samples from Market B did not contribute much to the variability of both PCs; the pesticide concentration levels were neither highest nor lowest. It can therefore be concluded that cabbage is more susceptible to pesticide bioaccumulation than the potatoes and Swiss chard.

Various correlations were observed between pesticide residues, which were also confirmed using cluster analysis. For example, a strong positive correlation was observed between DDT and its degradation product, DDD at *R* ^2^ = 1.00 while endrin and its degradation product, dieldrin were at *R* ^2^ = .97. Heptachlor was also positively correlated with its degradation products, chlordane, and heptachlor epoxide at *R* ^2^ = .58 and .12, respectively. Expectedly, the three chlors (heptachlor, chlordane, and heptachlor epoxide) were the only pesticide residues with a negative response to PC1 and their concentrations were highest in cabbage samples (Figure 1). However, negative correlations were also observed notably that between methoxychlor and other chlors with the heptachlor–methoxychlor relationship at *R* ^2^ = –.63 and between chlorbicyclen and other chlors with the chlordane–chlorbicyclen relationship at *R* ^2^ = –.65. These correlations between specific pesticides should be considered when doing bioaccumulation and health risk assessment studies.

### 3.4 Health risk assessment

The estimated daily intake and hazard quotients of the pesticides due to the consumption of the vegetables (cabbage, Swiss chard, and potato) are shown in Table 3. The risk assessment results of this study in which the hazard quotient (HQ) were less than one ranging between 2.04% × 10%^−3^% (hexachlorobenzene in Swiss chard) and 0.27% (heptachlor in cabbage samples) suggested that there was no health risk from the consumption of all the vegetables from the Bloemfontein markets. Although the pesticide residues were detected in the vegetables assessed in this study, the levels were still low and pose no risk to consumers. This, however, does not exclude the possible health risk due to consumption of vegetables from the same sources in future. Organochlorine pesticide residues are persistent and continuous application of such pesticides can lead to higher pesticide residue levels in the soil and subsequent uptake by plants which possibly will ensue in pesticide residue levels causing health hazards to be manifested (Taiwo, 2019). Elsewhere within South Africa, some of the organochlorines have been found to be associated with cancer risks, notably aldrin and dieldrin in vegetables of the KwaZulu natal Province (Buah‐Kwofie et al., 2019), DDT and hexachlorobenzene in vegetables around Johannesburg markets (Nuapia et al., 2016). Across Africa, the presence of pesticides in vegetables has been found to be of potential health risk to consumers in Togo (Kolani et al., 2016) and the DR Congo (Nuapia et al., 2016) while in Burkina Faso, the vegetables had no risk to the consumers (Lehmann et al., 2017).

**TABLE 3:** Health risk estimation for chronic effects associated with the highest pesticide residues

Pesticide residue	ADI (mg/kg)	Cabbage			Swiss chard			Potato		
		C_A_ (mg/kg)	EDI (mg kg/day)	HQ (%)	C_A_ (mg/kg)	EDI (mg kg/day)	HQ (%)	C_A_ (mg/kg)	EDI (mg kg/day)	HQ (%)
o.p'‐DDD	0.01	8.62E–06	3.38E–08	0.00034	9.1E–06	3.56E–08	0.000356	8.42E–06	3.3E–08	0.00033
p.p'‐DDE	0.01	8.18E–06	3.2E–08	0.00032	8.32E–06	3.26E–08	0.000326	9.26E–06	3.63E–08	0.000363
o.p'‐DDT	0.01	8.62E–06	3.38E–08	0.00034	9.1E–06	3.56E–08	0.000356	8.62E–06	3.38E–08	0.000338
Aldrin	0.0001	1.01E–05	3.94E–08	0.0394	9.28E–06	3.63E–08	0.036347	6.23E–06	2.44E–08	0.024381
Endrin	0.0002	7.55E–06	2.96E–08	0.01479	8.98E–06	3.52E–08	0.017576	8.43E–06	3.3E–08	0.016515
Dieldrin	0.0001	7.4E–06	2.9E–08	0.02898	nd	–	–	8.43E–06	3.3E–08	0.033031
Endrin aldehyde	0.0002	nd	–	–	nd	–	–	nd	–	–
Chlordane	0.0005	1.17E–05	4.57E–08	0.00914	2.9E–05	1.13E–07	0.022678	9.12E–06	3.57E–08	0.007144
Chlorbicyclen	0.0006	1.08E–05	4.21E–08	0.00702	nd	–	–	1.1E–05	4.32E–08	0.007202
Heptachlor	0.0001	7.02E–05	2.75E–07	0.27485	2.09E–05	8.19E–08	0.081858	1.95E–05	7.65E–08	0.076532
Heptachlor epoxide	0.0001	1.25E–05	4.88E–08	0.0488	1.1E–05	4.32E–08	0.04324	0.000013	5.09E–08	0.050917
Methoxychlor	0.1	7.17E–05	2.81E–07	0.00028	9.01E–05	3.53E–07	0.000353	9.15E–05	3.58E–07	0.000358
Endosulfan ether	0.006	1.23E–05	4.81E–08	0.0008	7.96E–05	3.12E–07	0.005193	6.72E–05	2.63E–07	0.004384
α‐Lindane	0.005	5.35E–06	2.1E–08	0.00042	nd	–	–	7.9E–06	3.09E–08	0.000619
Hexachlorobenzene	0.005	3.08E–06	1.21E–08	0.00024	2.6E–06	1.02E–08	0.000204	7.85E–06	3.07E–08	0.000615
Dicofol	0.002	nd	–	–	nd	–	–	nd	–	–

Abbreviations: ADI, acceptable daily intake; C_A_, average concentration; EDI, estimated daily intake; HQ, hazard quotient, nd, not detected.

The current legislature in South Africa prohibits a few organochlorine pesticide residues, which were only listed in 2016 except for DDT and dieldrin (1983), and chlordane (2005) (DAFF, 2017). It is appreciated that the Department of Agriculture, Forestry and Fisheries has pesticide management policy derived from South African Fertilizers, Farm Feeds, Seeds and Remedies Act No. 36 of 1947 that defines protocols for proper handling of pesticides, and human and environmental protection (DAFF, 2010). However, various limitations are present in the Act including (i) the lack of the country's pesticide monitoring program and registration; (ii) the penalty for breaching the regulations, which are not prohibitive enough to enforce compliance. This can prompt farmers to free use the banned and restricted pesticides without fear of legal consequences. There is therefore the need to revise the regulations of the Fertilizers Act to reflect the current international laws on pesticides management.

## 4 CONCLUSIONS

The present study evaluated the presence of the selected pesticide residues in staple vegetable samples, namely *Brassica* *oleracea* *var*. *capitata* (cabbage), *Beta* *vulgaris* *var*. *cicla* (Swiss chard), and *Solanum* *tuberosum* (potato), collected from different fresh produce markets in Bloemfontein City, South Africa. The use of QuEChERS and gas chromatography with high resolution mass spectrometer has allowed us to detect pesticide residues in the ng/kg scale well below the maximum residue limits in food sources. Using residue/by‐product ratios, it was predicted that input into the environment was from recent sources. Most of the targeted pesticide residues are not controlled in South Africa. While the concentrations of targeted pesticide residues were all below MRLs and posed limited health impacts, continued application of products containing these residues should be of concern. This is possible in the sense that some pesticides are persistent organic pollutants; therefore, continuous application of these pesticides can lead to high levels of pesticide residue levels in the soil and subsequent uptake by plants with the possibility of rising to levels at which health risks will become a reality. More studies are needed to confirm the sources of pesticides. Only a few organochlorine pesticide residues are banned in South Africa, and there is no tight legislature to ensure total compliance to pesticide application regulations. These deficiencies or gaps need to be addressed to comply with the country's and global requirements.

## CONFLICT OF INTEREST

None.

## AUTHOR CONTRIBUTIONS

**Nthabiseng Motshabi:** Conceptualization (lead); Data curation (equal); Formal analysis (equal); Investigation (equal); Methodology (equal); Writing‐original draft (equal). **Somandla Ncube:** Data curation (equal); Formal analysis (equal); Methodology (equal); Writing‐review & editing (equal). **Mathew** **Muzi Nindi:** Resources (equal); Supervision (equal); Writing‐review & editing (equal). **Zenzile Peter Khetsha:** Conceptualization (equal); Resources (equal); Supervision (equal); Writing‐review & editing (equal). **Ntsoaki Joyce Malebo:** Conceptualization (lead); Funding acquisition (lead); Project administration (equal); Resources (equal); Supervision (lead); Writing‐review & editing (equal).

## Data Availability

Data available on request from the authors.
